# [Corrigendum] ETS-1: A potential target of glycolysis for metabolic therapy by regulating glucose metabolism in pancreatic cancer

**DOI:** 10.3892/ijo.2026.5954

**Published:** 2026-11-02

**Authors:** Xiu Zhang, Dan Wu, Mohanad Aldarouish, Xiaodong Yin, Chunyan Li, Cailian Wang

Int J Oncol 50: 232-240, 2017; DOI: 10.3892/ijo.2016.3770

Following the publication of the above article and an Expression of concern statement (doi: 10.3892/ijo.2025.5786) that was published after a concerned reader drew the Editor's attention to the fact that, regarding the western blots shown in Figs. 4B and 5B, the AMPK panel in Fig. 4B looked strikingly similar to the ATG5 panel in Fig. 5B, and the p-AMPK panel in Fig. 4B looked highly similar to the ATG7 panel in Fig. 5B, the authors have now replied to the Editorial Office to address this issue. After carefully reviewing these figures, the authors have realized that the western blot panels from Fig. 5B were inadvertently duplicated and pasted into Fig. 4B, which resulted in the visual similarity between these two figures in the published paper. This error arose during the multiple rounds of revisions and adjustments, which led to an unintended copy-pasting error.

The revised version of Fig. 4, now showing the correct data for the AMPK and the p-AMPK blots in Fig. 4B, is shown opposite. The authors regret that these errors were not identified prior to the publication of the article, and thank the Editor of *International Journal of Oncology* for allowing them the opportunity to publish this corrigendum. All the authors agree with the publication of this corrigendum, and apologize to the readership for any inconvenience caused.

## Figures and Tables

**Figure 4.**
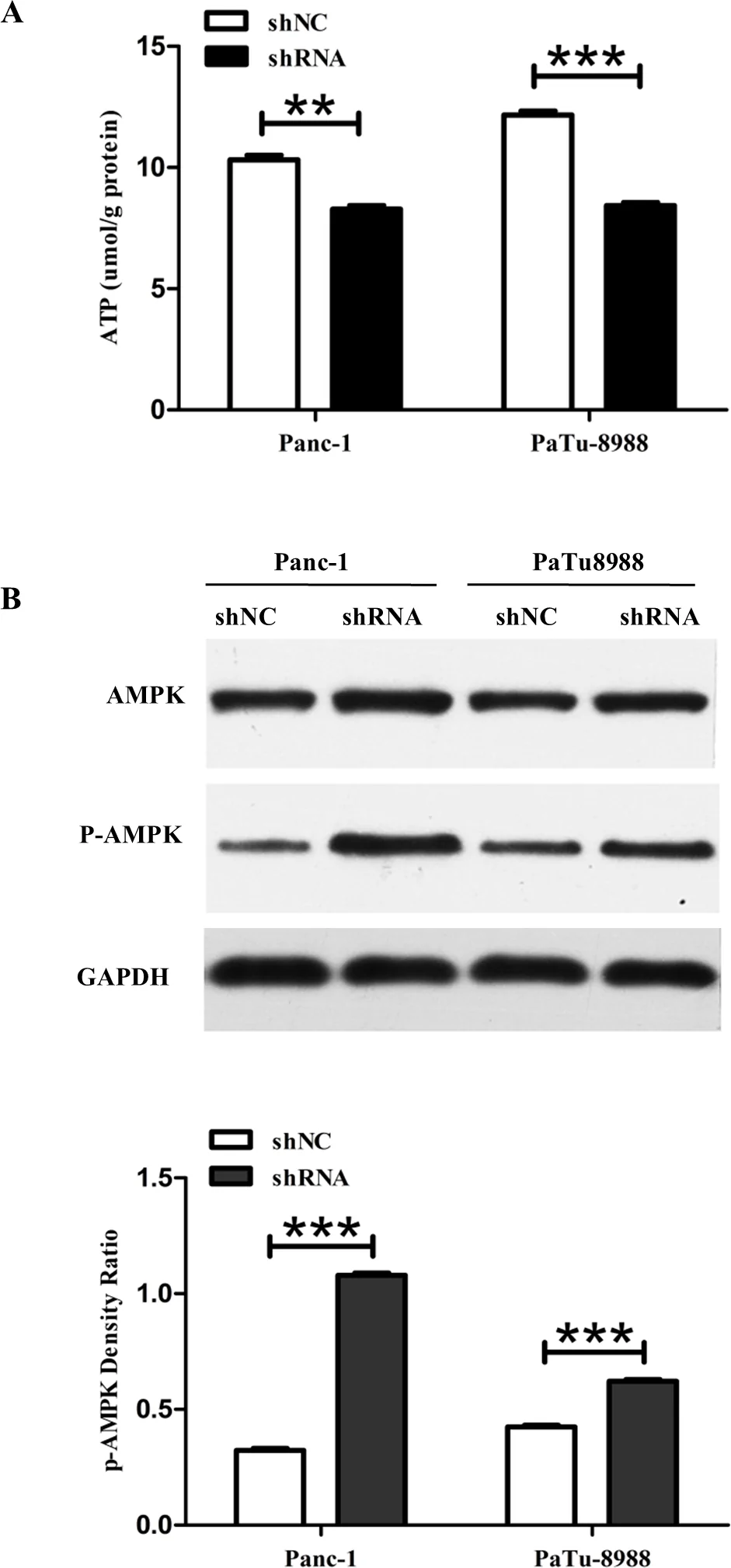
The ATP production and expression of p-AMPK protein in pancreatic cancer cells. (A) The ATP levels were detected by standard colorimetric

